# Managing stress in sports: the Pro•Stress program

**DOI:** 10.3389/fpsyg.2026.1826419

**Published:** 2026-07-29

**Authors:** A. Rui Gomes, Liliana Fontes, Clara Simães, Catarina Morais

**Affiliations:** 1 Psychology Research Centre, School of Psychology, University of Minho, Braga, Portugal; 2 Adaptation, Performance, and Human Development Research Group, School of Psychology, University of Minho, Braga, Portugal; 3 School of Nursing, University of Minho, Braga, Portugal; 4 Health Sciences Research Unit: Nursing (UICISA: E), University of Coimbra, Coimbra, Portugal; 5 Research Centre for Human Development, Faculty of Education and Psychology, Universidade Católica Portuguesa, Porto, Portugal

**Keywords:** anxiety, cognitive appraisal, intervention, performance, sport, wellbeing

## Abstract

Stress in sports has been a major topic of interest, both in terms of research and practical applications. Undeniably, stress can be a decisive factor for athletes’ performance and well-being. Thus, those who have the skills to effectively deal with it are much more likely to perform successfully and to positively adapt to stressful situations. However, gaps remain in the literature, namely how researchers and practitioners can help athletes to better manage and deal with the inevitable stressors they will face. This paper addresses these gaps by introducing and detailing the Pro**•**Stress program, based on the Interactive Model of Human Adaptation to Stress (IMHAS), which aims to promote stress management skills. With a strong theoretical background, this program is structured around the IMHAS and trains participants in dealing with stressor characteristics, improving cognitive appraisal, and promoting positive reactions, thereby leading to more positive adaptative results. This, in conjunction with applied training exercises and highly individualized tutoring, makes Pro**•**Stress a sound, well-founded option for those interested in stress management.

## 1 Introduction

The topic of stress has been on the forefront of research in any settings where humans must perform to very exacting standards, from academic to business contexts. Its modern scientific study began in the late 19th century (Robinson, 2018) and has spawned a myriad of publications regarding its concept, consequences, and how we humans can adapt to it. One of the first theories to explain stress was Cannon’s flight or fight response (Cannon, 1929 as cited in Robinson, 2018), which remains to this day one of the most influential works in stress research. According to the author, when faced with physical emergencies or psychological stress, the body assumes a response of either fleeing the situation or fighting. These are made possible through physiological changes, such as the release of adrenalin. More recently, researchers have included one more possible response: freezing (e.g., Roelofs, 2017). Building on this model, Selye (1936, as cited in Robinson, 2018) focused on describing how human bodies react to stress and, crucially, what happens when people are subjected to long-term stress, i.e., burnout. In his work, Selye (1975) distinguishes between the concepts of eustress and distress: the former refers to adaptations that are experienced as agreeable or beneficial, while the latter refers to the opposite.

There is one more example of early scientific research on stress worthy of mentioning: the work of Yerkes and Dodson (1908, as cited in Robinson, 2018) which resulted in the Yerkes-Dodson Law: in general, performance increases along with psychological and physical arousal, but only up to a point; when arousal becomes too high, performance levels decrease; when plotted on a chart, the Law takes the shape of an inverted *U*. Although today outdated, this research triggered many other works on this topic, thereby occupying an important place in the construction of our knowledge on stress.

World War II only increased interest in the topic, especially due to the high levels of stress both the military and civilian populations had to endure. It is in this context that arguably the most famous transactional theory of stress arose, Lazarus and Folkman’s (1984) Cognitive-Motivational-Relational Theory. Lazarus argued that stress is more than just its biological components; in fact, the author goes so far as to argue that the psychological dimension is even more important than the situation itself. It should be noted that the psychological dimensions include behaviors, cognitions, and emotions. Although highly interrelated and frequently presenting together, it is important to distinguish between these dimensions, in order to understand which is more prevalent and/or is more important to the individual’s experience: behaviors include what people do/how they act, cognitions refer to how/what people think, and emotions can be described as what/how people feel (Dolan, 2002). All are interconnected: what we do influences how we think and how we feel, what we think influences what we do and how we feel, and what we feel influences what we do and what we think. Lazarus and Folkman (1984) suggest the concept of cognitive appraisal as central to the understanding of stress: the way people interpret situations is what determines a successful adaptation to the event. However, this focus on cognition was not sufficiently debated at the time. Lazarus’ work spanned several decades, with a deep impact in research in all areas related to stress. In this same line of reasoning, the Biopsychosocial Model of Challenge and Threat (Blascovich, 2013) also highlights the processes of cognitive appraisal as central to adaptation to stress. Blascovich (2013) proposes that, when faced with a performance-related activity, individuals assess the demands of the situation and the resources they possess to deal with it. From this evaluation, people may experience a state of challenge, which occurs when resources are higher than demands, or a state of threat, which occurs when demands are higher than resources. This model also predicts different physiological responses depending on whether the person is in a challenge or threat state. In sum, this model posits that intervention in stress (regardless of context) must focus on reframing the situation’s demands and/or increasing people’s resources to deal with the demand. This will allow the person to reach a state of challenge, which promotes performance and well-being.

The context of sports is no different, with interest in the topic of stress beginning since at least the early 20th century, even though formal scientific research can be traced only to the late 20^th^ century (Martín-Rodríguez et al., 2024). Generally, the focus has been mainly on how stress influences performance. Particularly, researchers have been interested in understanding why stress can, simultaneously, hinder and facilitate performance, meaning that some athletes appear to benefit from stressful situations, achieving outstanding performances, while for others stress is debilitating and highly contributes to failure (Hancock and Hancock, 2014). Sports psychology researchers are thus highly invested in understanding this relationship and, crucially, in how to improve adaptation to stress in athletes. The question remains: how can we, researchers and practitioners, lead athletes to a situation where they can manage and deal with the inevitable stressors they will face? Although research in this topic is plentiful, we have yet to reach a consensus on how best to achieve this goal. Intervention programs typically include some form of Cognitive Behavioral Therapy, mindfulness and meditation techniques, mental skills training, among others (Kumar, 2025). This paper outlines an intervention program on stress management for athletes, informed by a conceptual model, with the objective of improving performance and well-being.

### 1.1 Stress in sports

#### 1.1.1 Stress in sports: current knowledge

Stress is characterized as a complex psychobiological process involving stressors, perceptions of danger, and emotional responses, such as anxiety (Spielberger, 2021). In sports, this complex issue profoundly impacts athletes’ performance and mental well-being. Therefore, it is imperative to reach a comprehensive understanding of the nature of stress and its implications for athletes, coaches, and sports organizations.

Recent research emphasizes the interaction among stress, anxiety, and performance, highlighting the necessity for effective stress-management strategies (Tossici et al., 2024). For example, anxiety, a typical emotional response to stressful situations, may negatively influence performance, and is linked to both injury prevention and rehabilitation (Ford et al., 2017). Additionally, mental fatigue, which stems from sustained cognitive exertion, can adversely affect both physical and mental performance (Loch et al., 2020). In order to mitigate these negative effects, it is crucial to implement mental recovery strategies, such as psychological techniques and sufficient sleep (Loch et al., 2020). The Revised Theory of Challenge and Threat States in Athletes, for example, suggests that perceiving stress as a challenge rather than a threat can enhance performance and psychological well-being (Silva and Farias, 2023). In this way, effective stress management is essential for the prevention of mental health issues such as burnout and anxiety (Nurjannah et al., 2022). Research indicates that effective stress management techniques can enhance athletes’ performance and overall organizational success (Nurjannah et al., 2022). While emphasizing stress and anxiety in sports remains important, it is also crucial to acknowledge the potential for resilience and growth through these challenges, as athletes may cultivate coping strategies that improve their performance and well-being over time.

#### 1.1.2 Stress in sports: programs and intervention domains

Stress in sports is a complex issue that requires specialized programs and interventions to effectively address the diverse stressors athletes encounter and to enhance their performance and well-being (Fitria et al., 2025). These interventions may be grouped into cognitive-behavioral strategies, psychological counseling, and structured support systems, each targeting different facets of stress management. These interventions use stress management techniques that include methods such as relaxation training, visualization, and mindfulness, which are commonly used to help athletes cope with stress. These approaches target autonomic, somatic, and cognitive stress responses (Suinn, 2005). Additionally, Cognitive Behavioral Therapy (CBT) effectively boosts mental resilience and helps manage stress, especially during competitive events (Fitria et al., 2025). Structured psychological intervention programs that incorporate mental health strategies into training can substantially enhance athletes’ recovery from injuries and diminish the likelihood of future injuries by effectively managing emotional stress (Fitria et al., 2025). Finally, support systems, encompassing family members, coaches, and teammates play an essential role in delivering emotional assistance, thereby potentially improving recovery and performance (Fitria et al., 2025).

All of these strategies have been associated with decreases in anxiety, better emotional control, and more adaptive responses to stress (Alkasasbeh et al., 2026). Some authors have found differences between sports contexts and populations. For example, intervention in psychological factors appears to be crucial in managing pre-competitive anxiety, but less so in unpredictable contexts. Moreover, any gains are highly dependent on the athlete’s motivation, awareness, and willingness, which vary immensely across ages and competitive levels (Alkasasbeh et al., 2026). Interventions based on mindfulness are increasingly common, encouraging attentiveness to the athletes’ present emotions and acceptance without attempting to control them. These appear to be effective in teaching athletes the skills to deal with inevitable competitive stressors. However, they do not always translate into objective performance gains (Alkasasbeh et al., 2026). Finally, psychophysiological and technology-assisted interventions, such as those based on biofeedback, have been increasing, with the objective of increasing athletes’ awareness of their own bio signals and, consequently, increasing their ability to control them (Alkasasbeh et al., 2026).

One of the most well-known intervention programs is proposed by Smith and Smoll (2002), which aims to reduce performance anxiety and burnout in young athletes, focusing on positive reinforcement techniques, encouragement, corrective instruction, and supportive communication. Studies on this intervention have garnered very promising results, with athletes showing decreased anxiety, more enjoyment, higher team cohesion, and higher self-esteem. More recently, other authors have proposed interventions based on the Rational Emotive Behavior Therapy (REBT), with a strong focus on the processes of cognitive appraisal: psychological distress is viewed as highly dependent of people’s interpretations, namely their irrational beliefs (Turner, 2014). One of such examples is the “Mindset: performing under pressure” program described as a multimodal cognitive-behavioral intervention to enhance stress mindset and reduce the irrational beliefs of athletes (Mansell et al., 2024).

Regarding the effectiveness of interventions, a systematic review identified several psychosocial interventions, including cognitive, multimodal, and alternative strategies, that optimize athletes’ stress experiences and performance outcomes (Rumbold et al., 2012). The effectiveness of these interventions depends on design features such as the type of treatment and the specific stress components addressed (Rumbold et al., 2012). While these interventions are crucial for effectively managing stress in sports, it is equally important to acknowledge that certain athletes may encounter stressors beyond their control, such as personal life challenges or organizational pressures, which can hinder their capacity to cope adequately (Arnold and Fletcher, 2021). Therefore, principal methodologies encompass CBT, stress management initiatives, and goal-setting techniques, which have been evidenced to enhance mental resilience and support recovery from injuries (Fitria et al., 2025).

In sum, athletes face a spectrum of internal and external stressors that can influence their performance and overall psychological well-being (Arnold and Fletcher, 2021; Suinn, 2005). Consequently, it is imperative to incorporate mental health strategies into training and rehabilitation regimes to effectively manage competitive pressures and emotional strain, thereby fostering a holistic approach to athlete care (Fitria et al., 2025; Rumbold et al., 2012).

#### 1.1.3 Stress in sports: what’s left to know?

Managing stress in sports remains an essential domain of scholarly inquiry, given that athletes encounter distinct pressures that influence their performance and overall well-being. In competitive sports, they face numerous stressors, such as competitive pressures, organizational demands, and personal expectations (Rumbold, 2014). If these stressors are not managed appropriately, they may result in adverse outcomes, highlighting the importance of implementing tailored interventions (Rumbold, 2014). Although numerous strategies have been investigated, considerable knowledge gaps persist regarding effective stress-management approaches specifically designed for competitive settings. Subsequently, we delineate critical elements of existing understanding and highlight areas requiring further research. Innovative strategies, including yoga, aerobic exercise, and holistic treatments such as acupuncture, have been demonstrated to improve resilience and reduce anxiety (Jain et al., 2023). Nonetheless, psychosocial interventions remain crucial as they assist athletes in stress management and promote overall well-being (Bhadauriya and Tripathi, 2018).

Concerning the role of psychological support, understanding athletes’ experiences of stress can inform the development of effective organizational stress management interventions (Rumbold, 2014). Case studies, such as Rebecca Adlington’s experience during the Olympics, exemplify the psychological challenges faced by athletes and underscore the importance of coping strategies (Devonport, 2015). Thus, despite advancements in stress management techniques, the necessity for a more robust evidence base and customized interventions persists. Future research should emphasize the long-term impacts of stress management strategies and their suitability across various sports and competitive tiers. Considering the need for providing systematic programs of stress management that can be applied in distinct stressful activities and then tested in terms of efficacy, this paper presents the structure and purpose of the Pro**•**Stress Program.

## 2 The Pro**•**Stress program

### 2.1 Theoretical background

The Pro**•**Stress Program is a cognitive-behavioral intervention based on transactional and interactive models of human adaptation to stress. Specifically, the Cognitive-Motivational-Relational Theory of Lazarus and Folkman (1984), Lazarus (1991, 1999) reinforces the processes of cognitive appraisal of stress, describing primary cognitive appraisal (threat and challenge) and secondary cognitive appraisal (coping) as central concepts to understand how people evaluate and cope with stress. Taking this perspective as background, the Interactive Model of Human Adaptation to Stress (IMHAS; Gomes, 2014), also points out cognitive appraisal as a pivotal dimension to understand how people adapt to stress, but also recognizes the need to understand the characteristics that can turn an event into a stressful one, the need to better detail primary cognitive appraisal by considering the “importance perception” dimension to explain why some individuals appraise the same situation as stressful while others do not evaluate it in the same way (“trigger” of stress adaptation), the need to better detail the secondary cognitive appraisal by considering the role of control perception regarding the stress event, and the need to incorporate in the stress adaptational process the triple levels of human reactions at psychological, physiological, and behavioral level (Gomes, 2014). It is quite interesting that transactional models of adaptation to stress are quite well-known and are used to explain how athletes evaluate and cope with sports demands (Nicholls et al., 2016; Nuetzel, 2023), but there is a scarcity of findings of how these proposals can contribute to improving the athletes’ ability to cope with stress and eventually achieve higher levels of performance and wellbeing during their careers. In this way, the Pro**•**Stress Program specifically addresses this gap in literature by training athletes in the core dimensions of transactional perspectives, trying to give them an opportunity to improve the way they evaluate and cope with stress.

**Figure 1:**
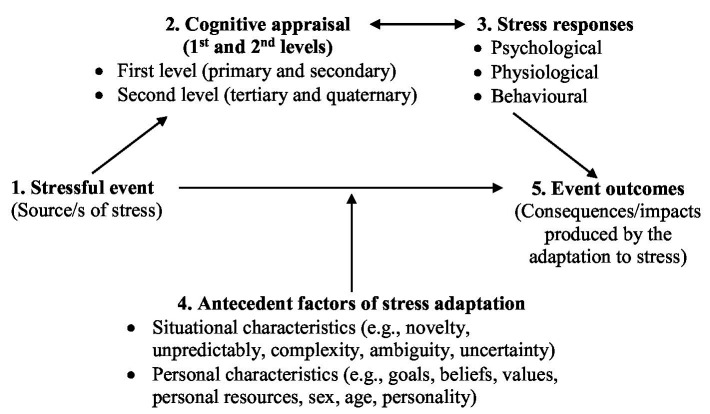
Interactive model of human adaptation to stress.

The Pro**•**Stress Program is directly related to the Interactive Model of Human Adaptation to Stress (IMHAS; Gomes, 2014), encompassing the same emphasis on the stressful event, the cognitive appraisal processes, and the individual responses to stress (see Figure 1). The stressful event can be described in relation to five dimensions (novelty, unpredictability, complexity, ambiguity, and uncertainty). Higher levels of each may negatively impact the subjective experience of stress. Cognitive appraisal is central to the model, beginning with importance perception, referring to the significance that the person attributes to the event, in regard to his/her well-being (“Is the stressful event relevant for me?”). Then comes threat perception, referring to feelings of harm or potential loss attributed to the event (“What negative consequences can arise from this stressful event?”), and challenge perception, referring to difficult-to-attain, yet anticipated gains attributed to the event (“What positive consequences can arise from this stressful event?”); these three factors are part of the primary cognitive appraisal. Also important, the model includes secondary cognitive appraisal processes, constituted by the dimensions of coping perception, which refers to the resources the person has to manage the stressor (“What can I do regarding this stressful event?”), and control perception, referring to the level of personal control that the person has regarding the stressful event and ways of dealing with it (“Can I control this stressful event or does it control me?). Ideally, in order to successfully adapt to the situation, it should be perceived as moderately important, with the lowest possible threat and the highest possible challenge, coping, and control perception. In the IMHAS, the primary cognitive appraisal (importance) is the gateway to whether people experience stress or not. If an event is seen as unimportant to the person, the appraisal process may stop and there is less probability of mobilizing the cognitive processes of challenge, threat, coping, or control. Thus, individuals must attribute some importance to the event so that they can gather resources and mobilize them to deal with the situation. For example, an athlete competing in a minor event after competing in the Olympics may see that competition as unimportant, and thus is less likely to be motivated to employ efforts to positively adapt to the situation. Another important distinct aspect of the IMHAS is the acknowledgement that processes of cognitive appraisal can occur at first level (i.e., when the person has to deal with a stressful event that is determined in terms of time and circumstances) and second level (i.e., when the person has to deal with a stressful event that is not determined in a specific time and circumstance, eventually becoming repetitive and chronic).

Finally, how people appraise the situation largely determines their reactions to stress, which we categorize as psychological (what people think), physiological (how people feel), and behavioral (how people react immediately to the stressful event). This is a looped process: people’s reactions will impact the following cognitive appraisal processes. Depending on people’s assessment of the stressor, their cognitive appraisals, and their reactions, the final adaptation to stress may be positive or negative.

The Pro**•**Stress program, described in the following sections, is structured around the IMHAS (Gomes, 2014), includes practical exercises based in the model, and culminates with a stress management plan that each person formulates to apply to a specific stressful situation. Also important, the training of stress management respects the principles of life skills training (Gomes and Resende, 2020), mainly in aspects related to the four stages of life skills learning and acquisition: (a) motivation (e.g., participants are encouraged to analyze how stress management can be a useful tool for their lives), (b) learning (e.g., participants are introduced to the main factors of stress management as established by the IMHAS), automatization (e.g., participants are trained on the main factors of stress management as established by the IMHAS and should be able to assume strategies to improve their ability to deal with stressful events), and transference (e.g., participants delineate and assume a stress management plan that can be applied in distinct areas or aspects of their lives).

To encourage participants’ progression through the program, we use four main teaching/training methods:

1. Expository: transmit information about the stress management life skill.
2. Independent reflective work: deliver individual activities to stimulate reflection on the stress management life skill.
3. Collective reflective work: deliver group activities to stimulate reflection on the stress management life skill.
4. Simulated skills work: deliver group activities carried out by participants to stimulate the acquisition of the stress management life skill.
5. Self-referenced skills work: deliver group activities carried out to stimulate the application of the stress management life skill in one or more areas of each participant’s life.

### 2.2 Application conditions

The Pro**•**Stress program can be used in-person and via remote learning. If in-person, it requires a room for 15 people, with chairs and tables, a projector, and an internet connection.

The program functions in groups numbering between 8 and 12 participants to ensure that all receive individualized tutoring during the sessions. Adolescents and adult participants from distinct areas of human functioning are welcome to the program. The program is geared toward participants with any educational and professional background. All different types of professions are welcome, although there is the possibility of delivering the program to one group of only one profession (e.g., athletes), which brings some advantages and disadvantages. On the one hand, different backgrounds enrich the program’s sessions and may help participants acquaint themselves with different ways of thinking. On the other hand, one homogeneous group ensures that everyone’s experiences will resonate with each other’s, thereby facilitating the discussion of individual stress management plans. To accommodate people from distinct areas of living, the program includes examples from sports, but also from other contexts, as is the case of educational, health, business, military, among others. This means that activities can be selected to fit each context while maintaining the same topic of training and congruency of intervention.

The program comprises 45 h of synchronous 3 h sessions (15 in total) with two psychology professionals (“monitors”) at all times. Due to the psychological theoretical framework of the program, psychologists may be the professionals better prepared to deliver the program, although other professionals of human sciences may also deliver the intervention, since they have the knowledge and training to intervene in human adaptation to stress following transactional and interactive perspectives.

Before the program starts, the participants are assigned to one of three teams that will remain the same throughout the program—although some of the work is individual, there are many small-group exercises. When preparing these exercises, each team goes to a different room. During this time, the two monitors oversee all activities and help all teams as needed.

### 2.3 General structure

The time structure is somewhat flexible and may be tailored to the participants’ requirements. The 15 sessions include evaluation (pre-intervention, post-intervention, and follow-up), presentation of the program, the three components of the model (stressors, cognitive appraisal, and reactions), formulation of a stress management plan, and its training. Table 1 summarizes the program’s organization. As can be seen in this table, the training related to stress characteristics takes around 12 h (Part 2), cognitive appraisal takes around 18 h (Part 3), stress reaction takes around 6 h (Part 4), and the final training of the stress management plan takes around 9 h (Part 5). This division has the advantage of allowing the monitors to design specific interventions directed to one or more parts of the program, although all of them are theoretically involved in the way athletes evaluate, cope, and react to stressful events. Nevertheless, this flexibility may make the application of the program more realistic when there are evident time, resources, or opportunity constrains. For example, a sport psychologist may find it particularly useful to give special attention to stress reactions training to the athletes before the final playoffs of the competitive season (opportunity constrain). This also may allow the sport psychologist to divide the program along the sport season; for example, training in stress characteristics can be provided in the beginning of the season (allowing athletes to better understand the kind of stressors that they may have to face), then, after competition begins, the sport psychologist can focus the training on positive and functional patterns of cognitive appraisal, and, as mentioned, the training of stress reactions can be provided before key moments of the sport season.

**Table 1:** Pro**•**Stress program: organization and areas of training.

IMHAS	Pro•Stress program
Evaluation	Part 1. Pre-intervention evaluation
	Goals
	To determine the participants’ stress management skill level before intervention
	Duration: 15 min (before session 1)
Program presentation	Presentation
	Goals
	Present the program’s goals and main activities
	Assemble the participants’ teams
Stressor characteristics	Part 2. Stressor characteristics
Novelty	Goals
Unpredictability	To teach the definition of stressor event and its characteristics (novelty, unpredictability, complexity, ambiguity, and uncertainty), how to identify them and manage them
Complexity	To train participants in how to deal with stress characteristics
Ambiguity	To begin writing a stress management plan, according to the stressor characteristics
Uncertainty	Duration: 4 sessions; 3 h per session (12 h)
Cognitive appraisal	Part 3. Cognitive appraisal
Importance	Goals
Threat	To teach the cognitive appraisal of stressors and its components
Challenge	To train participants in how to establish positive patterns of cognitive appraisal
Coping	To continue writing a stress management plan according to each participant’s level of cognitive appraisal dimensions
Control	Duration: 6 sessions; 3 h per session (18 h)
Reactions to stress	Part 4. Reactions to stress
	Goals
	To teach the reactions to stress and their components
Psychological	To train participants in how to control the stress reactions
Physical	To adjust the stress management plan according to each participant’s level of reactions to stress
Behavioral	Duration: 2 sessions; 3 h per session (6 h)
	Part 5. Training of the stress management plan
	Goal
	To assume the strategies of stress management defined in simulated situations
	Duration: 3 sessions; 3 h per session (9 h)
Evaluation	Part 6. Post-intervention evaluation
	Goals
	To determine the participants’ stress management skill level immediately after intervention
	Duration: 15 min (after the last session)
Evaluation	Part 7. Follow-up intervention evaluation
	Goals
	To determine the participants’ stress management skill level after intervention
	Duration: 15 min (3 to 4 months after the last session)
Total	Sessions: 15
	Duration: 45 h

### 2.4 Part 1: evaluation and presentation

Evaluation is fundamental to determine the efficacy of the Pro**•**Stress program. Participants complete self-report measures on their stress management skill level and its impact on their lives. This assessment allows us to establish if participants finish the program with a higher level of skill than when they started, thereby providing a measure of the program’s efficacy. Thus, participants complete this evaluation before the program starts, immediately after it ends, and in a follow-up period, two to four months later, to assess the eventual maintenance of expected changes. Each professional and researcher may choose the most appropriate measures to determine the program efficacy. However, in order to improve replicability across studies, it is important that measures to evaluate the program’s efficacy reflect, as much as possible, the main dimensions of the IMHAS model and, consequently, the intervention areas of the Pro**•**Stress program. Specifically, the first dimension refers to the stressful event of the IMHAS and stressor characteristics of the Pro**•**Stress; in this case, measures of general stress (Cohen et al., 1983) and stress in sports (Morais et al., 2025) are suitable, although it is possible that the latter are more recognized for athletes than the former. The second dimension is cognitive appraisal (both for IMHAS and Pro**•**Stress), for which several measures are available in literature, some of them used in sports (Morais et al., 2025; Rossato et al., 2018). The third dimension refers to stress responses (IMHAS) or reactions to stress (Pro**•**Stress), implicating typical reactions to stress should be evaluated, including the psychological/emotional, physiological, and behavioral reactions; some measures are available to evaluate these reactions (Connor-Smith et al., 2000; Debowska et al., 2022; Schlebusch, 2004). The event outcomes of the IMHAS (and consequences produced by the Pro**•**Stress program) should be selected according to the needs and interests of researchers and, in fact, the diversity of measure outcomes is welcome, because it would allow to evaluate the potential multiple impacts of the intervention program. Along with the development of the Pro**•**Stress program, a general measure of adaptation to stress named Stress Management Readiness Questionnaire (SMRQ; Gomes, 2020) is also being developed that captures all the dimensions of the IMHAS model and all areas of training of the Pro**•**Stress. The measure intends to evaluate stress management readiness, i.e., people’s mental representations of their potential for stress management, which translates into a tendency for the person to assume cognitive, emotional, and behavioral stress management skills. Whenever this potential is successfully put into practice in different everyday situations, it can be said that the person has mastered the skill of stress management when facing stressful situations. Thus, the SMRQ includes 18 items answered on a 5-point Likert scale assessing the characteristics of the stressful event (i.e., the adaptative stimuli), cognitive appraisal, and reactions/responses to stress. In sum, researchers interested in implementing the Pro**•**Stress have distinct measures to use in order to test the efficacy of the program.

The program is presented in the first session (included in Part 1; see Table 1), namely its goals, structure, and evaluation system. Participants are divided into three teams, for which they must choose a name and motto (this reinforces team identity and facilitates various training activities) (cf. Hoult et al., 2024; Slater and Barker, 2019).

### 2.5 Part 2: stressors

Part 2 is dedicated to stressors and their characteristics, the first dimension of the IMHAS proposal (Table 2). All three main parts of the program (stressors, cognitive appraisal, and stress reactions) follow the following structure: a theoretical exposition, a short knowledge test and practical exercises, ending with the formulation of the stress plan that will be complemented in Part 5.

**Table 2:** Stressor characteristics, definition, and mental plot.

Stressor characteristic	Definition	Mental plot
Novelty	The person is unfamiliar with the stressor or the stressor has properties and characteristics that are different from those previously experienced.	“This has never happened to me.”
Unpredictability	The stressor occurs in circumstances that are unexpected for the person (i.e., unexpected timing, context, and duration).	“I did not expect this to happen.”
Complexity	The stressor involves complex and demanding modes of functioning (cognitive and/or physical) for the person.	“This is very difficult!”
Ambiguity	The stressor lacks clear and objective criteria about what is expected of the person (i.e., desirable actions, expected performance, purpose of the event).	“I do not know what is expected from me.”
Uncertainty	The stressor does not allow the person to reasonably anticipate its impact on one’s well-being nor predict the final outcome (i.e., predictable performance or result, behavior or performance of others).	“What could happen?’

Our goals are to teach the dimensions of stressor characteristics (novelty, unpredictability, complexity, ambiguity, and uncertainty), how to identify and manage them, and to begin writing a stress management plan, according to the stressor characteristics participants identify in their own situations.

Specifically, we begin with a theoretical exposition to explain stress and stressors and then ask participant to test their knowledge by doing a short theoretical test with four multiple-choice questions with five response options, with a time limit of 5 min (stage 2 of life skills acquisition). Participants are allowed to consult the course’s materials during the test. The test is always corrected when participants finish, to ensure that this part’s content is well and truly established. Participants are encouraged to share and discuss their answers and rationale.

After discussing the stressor characteristics, participants are presented with several case analysis where they have to figure out which stress characteristic is most prevalent. This will reinforce the stage 2 of life skills acquisition (learning). Then, using the same cases, we ask participants how they can face and manage the stressor in that particular situation (stage 3 of life skills acquisition). This is discussed in the larger group with all participants in order to clarify that several strategies can be used to deal with the same stressor event. Lastly, the activity evolves into team roleplaying the situations and applying the strategies they brainstormed previously (stage 3 of life skills acquisition). For this roleplay, one team plays the case’s character, another team will interact with them, and the third team observes, looking out for strategies adequate to the case and for five desirable characteristics of the stress plan: observable, controllable, appropriate, realistic, and enthusiastic. We end this exercise with a large-group discussion—the teams reflect on their plan and execution, sharing challenges and ideas.

Our focus is on how to deal with each stressor characteristic, which will enable participants to start thinking about their own stress management plan and how they can deal with their stressors. Moreover, it allows this knowledge to be transferred to other situations—regardless of the scenario, the same principles apply (stage 4 of life skills acquisition).

Although there are many potential strategies to deal with the characteristics of stressors, we discuss with participants the ones presented below (this is also repeated in the following parts of the program).

#### 2.5.1 Novelty

- Anticipate possible stressors;
- Look for similarities between the new stressor and others that the person has successfully dealt with in the past;
- Act from the simplest to the most complex;
- If necessary, avoid confronting and managing the stressor when it occurs;
- Seek support and information about the stressor from credible sources.

#### 2.5.2 Unpredictability

- Anticipate possible stressors (when, where, and how do they tend to occur?);
- Start with strategies/solutions that have worked in the past;
- If necessary (and possible), avoid facing and managing the stressor when it occurs.

#### 2.5.3 Complexity

- Act from the simplest to the most complex;
- Seek support and information about the stressor from credible sources;
- Be in the best possible physical and mental conditions before facing the stressor.

#### 2.5.4 Ambiguity

- Seek to clarify exactly what is expected of the person and the stressor;
- Propose and/or adopt internal and controllable indicators of success.

#### 2.5.5 Uncertainty

- Prioritize and value internal and controllable indicators of success;
- Be in the best possible physical and mental condition before facing the stressor.

Although there are many potential strategies to improve the quality of stress management plans, we also discuss with participants the characteristics of these strategies, such as the ones presented below (this is also repeated in other parts of the program).

- *Observable*: strategies should be clear in terms of the impact in multiple domains of human functioning, including mental, physical, and behaviors to assume.
- *Controllable*: strategies should depend as much as possible on the participant’s capabilities and resources.
- *Appropriate*: strategies should be tailored to the participant’s stress event.
- *Realistic*: strategies should preferably have been used successfully in the past or, failing that, at least be likely to succeed.
- *Enthusiastic*: strategies should generate feelings of challenge and personal development.

Finally, it is important to mention that along the realization of these activities some electronic platforms are used during the intervention program to optimize the learning process and speed of delivering activities (the same happens in other parts of the program).

### 2.6 Part 3: cognitive appraisal

Part 3 is dedicated to cognitive appraisal, the second, and arguably the most important, component of the IMHAS proposal (Table 3). This part follows the same structure as Part 2: a theoretical exposition, a short knowledge test, and practical exercises, ending with the formulation of the stress plan that will be complemented in Part 5.

**Table 3:** Cognitive appraisal dimensions.

Cognitive appraisal dimension	Definition	Mental plot
Importance perception	The stressor is assessed as relevant in terms of the person’s values, beliefs, and principles in life.	“This is crucial.”
		“This is of no interest.”
Threat perception	The stressor is assessed as disruptive and negative to the person’s well-being and functioning.	“This is going to go badly!”
Challenge perception	The stressor is assessed as stimulating and exciting from a personal point of view.	“This is what I wanted most!”
Coping perception	Assessment that the person makes of their resources and abilities to deal with the demands of the stressor.	“I’m going to do this!”
Control perception	Assessment that the person makes about their decision-making power and control over the stressor.	“This does(n’t) depend on me!”

Our goals are to teach the dimensions of primary and secondary cognitive appraisals (importance, threat, challenge, coping, and control perceptions), how to identify and manage them, and to begin writing a stress management plan, according to the appraisal characteristics participants identify in their situation.

Specifically, we begin with a theoretical exposition to explain cognitive appraisal and its distinct dimensions and then ask participant to test their knowledge by doing a short theoretical test with four multiple-choice questions with five response options, with a time limit of 5 min (stage 2 of life skills acquisition).

During this theoretical exposition, there are some aspects that should be emphasized: cognitive appraisal includes those processes that identify how the stressor is assessed in terms of the person’s well-being (primary appraisal) and confronted in terms of the person’s cognitive and behavioral resources (secondary appraisal) (see Table 3). The first dimension, importance perception, relates to how relevant the person assesses the stressor to be. An evaluation of either too high or too low importance will have negative consequences in the adaptation to stress—if the event is seen as crucial, the person will likely feel more pressure, since any mistake will be seen as very threatening to the person’s values, beliefs, and principles. If, on the other hand, the stressor is seen as having no interest, it likely follows that the person will not feel motivated to act. Thus, typically, importance perception should be neither too high nor too low.

Threat and challenge perceptions are intimately connected and should not be seen as separate dimensions, because it is quite common for people to assess the stressor as having some degree of threat and some degree of challenge. Challenge appraisals occur when the stressor is viewed as stimulating and exciting. Nevertheless, the higher the challenge, the lower the threat and vice-versa. Thus, our goal is to maximize the challenge while minimizing the threat. Within the secondary appraisal, coping perception refers to the resources the person has to face the stressor, which should be as high as possible. Finally, control perception assesses how much control the person feels over the stressor, which should also be as high as possible.

In sum, cognitive appraisal is central to our program—one cannot change the stressor itself (although one can use strategies to deal with its characteristics), but one can definitely change one’s cognitive appraisal of the situation by, for example, calibrating importance to a moderate level, reducing threat perception while maximizing challenge perception, and increasing coping and control perceptions.

After presenting these dimensions, we challenge the participants to try and discuss strategies to deal with each one, in a short brainstorming activity involving all the group. We then present a few suggestions to complement theirs. These include:

1 How to manage *importance*:

- Understand the factors that can make the stressor unimportant or overly important;
- Create a positive personal meaning when the stressor is unimportant (e.g., set challenging goals that make the situation stimulating);
- View the stressor as ‘just’ another task in life, among all the other tasks and responsibilities of life, when the stressor is overly important.

2 How to manage *threat*:

- Understand the factors that are making the stressor negative and threatening to the person;
- Look for alternative ways to assess the negative and threatening aspects of the stressor (e.g., viewing them as a challenge);
- Change the negative and threatening aspects of the stressor (e.g., formulate action plans to reduce threatening aspects).

3 How to increase *challenge*:

- Analyze how the stressor can stimulate and increase personal capabilities;
- Set personal challenges for the stressor that depend on the person’s actions and capabilities.

4 How to increase *coping*:

- Analyze the demands placed by the stressor and check which personal skills are appropriate for dealing with the stressor;
- Apply personal strategies for dealing with the stressor that have worked in the past.

How to increase *control*:

- Analyze the extent to which aspects of the stressor can be controlled through the person’s actions;
- If possible, act on the aspects that depend most on the person’s actions and only then act on the more uncontrollable aspects.

Regarding coping strategies, we discuss with participants their skills, resources, and abilities to deal with the stressor event. Thus, we present five different coping resources that people can use when confronting their stressor. Table 4 summarizes each coping strategy. Not all coping resources are created equal—some are more adaptative than others, generally speaking. All of them can and should be used with different stressors. Typically, the most useful is problem solving, which includes those strategies that actually attempt to solve the problem directly. But problem solving may not be enough, since it does not deal with the emotional aspects of the situation. For that, one must use active and/or passive emotion regulation. The first is much more useful and adaptative, but there are very specific situations where active regulation is not possible or would not be adaptative. In situations where the person has very little control and/or in situations where the problem does not have a solution, for example, then passive emotion regulation can be used. These situations include, for example, incurable diseases, where denial may offer some protection to the self. Finally, the last two strategies focus on social support, either more emotional or more instrumental, with the latter having a more direct impact on actually solving the problem and the former working more directly in emotions regulation. Regardless of the situation, participants should look into these five different resources and utilize those that are more relevant to their situation.

**Table 4:** Type of coping strategies.

Coping resource type	Definition	Example
Problem solving	Strategies that seek to resolve the problem, its causes, and consequences.	Draw up action plans and implement them;
		Increase effort and concentration on tasks.
Active emotion regulation	Strategies that seek to reduce emotional distress resulting from exposure to stressors	Positive reappraisal of the stressor;
		Self-control and management of negative emotions.
Passive emotion regulation	Strategies designed to divert a person’s attention away from the stressor, causing them to think and act as if it were not happening	Denial;
		Self-blame.
Emotional social support	Strategies focused on seeking help from others in order to share how one feels about the stressor.	Sharing and venting negative emotions.
Instrumental social support	Strategies focused on seeking help from others in order to resolve the stressor.	Sharing opinions and alternatives for action.

After discussing coping strategies, participants complete a few practical group exercises, to enable them to apply their knowledge. The first activity includes case analysis aiming to help participants recognize and distinguish how stressors can be assessed (stage 2 of life skills acquisition). They are presented with five different situations where they must decide which cognitive appraisal dimension is more evident. This will help them characterize their own situations and, consequently, help guide their efforts to deal with them.

In phase 2 of this exercise of case analysis, participants reflect on how they can manage each stressor (stage 2 of life skills acquisition). They discuss within their teams which strategies can be used for each situation, which will be shared with the whole group in the final discussion. Finally, in phase 3, we present five cases that again highlight one of the cognitive appraisal dimensions. This time we add a few sentences referencing what the character did to manage the stressor. In teams, participants are asked which type of coping strategy is being used, from the five types discussed before. The three teams gather again at the end to discuss their answers (stage 2 of life skills acquisition).

By this point, we expect that participants will have a clear understanding of coping strategies. The last exercise of Part 3 attempts to test that knowledge in practice, albeit in a controlled and simulated environment (stage 3 of life skills acquisition). Still, we have found that participants enjoy this exercise and feel that it creates some tension, which gives them an opportunity to try and deal with it in a safe context. Specifically, the activity is very simple: to count certain letters in specific text lines riddled with spelling mistakes within a short period of time; the activity includes four tasks that increase in difficulty (participants have to count more letters in the same set period of time). The activity is done in front of others with some distracting feedback from monitors regarding the “excessive” time that they are using and possible errors in letter-counting made by the participants.

When all participants have completed phase 1 of the exercise, we have a group discussion and explain the rationale of the exercise, discussing how they felt and which strategies, if any, they tried to use. Participants are told that they will now repeat the task, with different letters, but now making an effort to use more adaptative coping strategies (phase 2 of the exercise). Participants are encouraged to favor problem-solving strategies, such as “Focus! Mark the letters as you count them.” Most immediately understand that their strategy should change according to the task’s complexity—so, for the first couple of tasks they can count letters at the same time, but when the number of letters increases to 3 and 4 it is better to count them separately. Then, we focus on emotional regulation strategies. Active regulation involves thoughts like “I can do this.” In this case, passive regulation may be helpful, and participants are encouraged to use statements such as “I cannot hear you! It’s very quiet in here!.” Finally, social support strategies are not as useful here, although we do suggest that they think about their family and friends and how they can count on them. Now that all participants have their own “mini-plan” to manage this stressor, the exercise is repeated, using different letters and lines. The instructors maintain the same distracting feedback and reminders about time. It is expected that the participants’ objective performance will improve, along with their subjective experience. So far, we have found that their performance is never worse, and actually improves most of the time. The participants’ subjective experience always radically improves—in a follow-up discussion, they typically mention feeling more confident and being more capable of tuning out our distractions. It is therefore a very useful exercise to ease people into applying stress management plans, in a controlled environment, along with showing them what their common reactions to stress are. Nevertheless, it should be said that this activity is delivered with the utmost caution so as to not represent a negative experience for participants. Thus, participants are advised they can leave the activity at any time if they are feeling too stressed and the levels of distracting feedback provided by monitors are delivered in a progressive way, meaning that if participants are experiencing difficulties in executing the task they receive less or even no distracting feedback by the monitors. Also important, the main goal of the exercise is allowing the participants to improve their ability to execute the task across different stages, meaning that the purpose is giving participants the opportunity to establish and test “mini-plans” for stress management and our experience with this exercise is that participants do improve their ability to manage this exercise along its repetitions. It should be reinforced that this exercise does not intend to put participants in a negative situation. Also, the monitors may choose to not apply this activity in the program, since it does not prevent the fundamental aspects of this part of the program, sufficiently explained by previous activities. Finally, we advise practitioners to only apply this activity when there is sufficient rapport between monitors and participants.

Part 3 ends with the development of the individual stress management plan (stage 3 of life skills acquisition). We focus on cognitive appraisal processes, which will enable participants to start thinking about their own stress management plan and how they can deal with their stressors. Moreover, it allows this knowledge to be transferred to other situations—regardless of the scenario, the same principles apply (stage 4 of life skills acquisition).

### 2.7 Part 4: stress reactions

Part 3 is dedicated to stress reactions, the third component of the IMHAS proposal (Table 5). This part follows the same structure as previous: a theoretical exposition, a short knowledge test, and practical exercises, ending with the formulation of the stress plan that will be complemented in Part 5. Our goals are to teach the dimensions of stress reactions (psychological/emotional, physiological, and behavioral), how to identify and manage them, and to begin writing a stress management plan, according to the reactions participants identify in their situation.

**Table 5:** Reactions to stress.

Reactions to stress	Definition	Example
Psychological | Emotional	Cognitive and emotional reactions resulting from exposure to stressors	Enthusiasm or discouragement, pessimism or optimism, sadness or joy, motivation or demotivation, commitment or disengagement, satisfaction or dissatisfaction, etc.
Physiological	Cardiovascular, biochemical, and gastrointestinal reactions resulting from exposure to stressors	Increased heart rate, sweating, stomach and intestinal problems, etc.
Behavioral	Approach or avoidance reactions to the stressor, resulting in feelings of success or failure during its occurrence	Impulse to act, avoidance of the situation, daily routines, etc.

Specifically, we begin with a theoretical exposition to explain stress reactions and distinct dimensions and then ask participant to test their knowledge by doing a short theoretical test with four multiple-choice questions with five response options, with a time limit of 5 min (stage 2 of life skills acquisition).

Specifically, we begin with a short activity where we ask participants to name different reactions to stress, which helps us to determine their level of knowledge, as well as to kick-start a discussion on the topic (stage 2 of life skills acquisition). Formally, we define reactions to stress as how a person feels in the face of a stressor (disruptive event) in three dimensions (see Table 5): psychological/emotional, physiological, and behavioral. Participants are asked to think in terms of how they think (psychological dimension), how they feel (physiological dimension), and how they act (behavioral dimension) when facing a stressor.

This classification helps participants to notice which reactions are more common for them, and to devise specific strategies for each dimension. It is important to note that, in their stress management plans, emotional reactions fall under the physiological dimension, in order to distinguish between the cognitive and emotional dimensions.

With this distinction clear, participants brainstorm ideas on how to promote positive reactions. Typically, participants already have some knowledge of relaxation and breathing techniques, which is helpful to transition into teaching them abdominal breathing techniques. Other common ideas include practicing yoga and mindfulness.

Before going into these techniques, we focus on the four A’s:

- To *A*ccept (and be aware of) the “normal” or “natural” reactions to exposure to stressors;
- To *A*nticipate the conditions that may facilitate or exacerbate the occurrence of stressors (personal and situational factors) and, if possible, to act on these factors.
- To *A*ct positively on reactions to the stressor (managing emotions and/or acting on the stressor) and to value functional changes in reactions felt in the face of the stressor.
- To *A*ssess the management of stress reactions, understanding what contributed to increasing and/or decreasing reactions to the stressor.

After this exposition, we proceed with the usual knowledge short test (part 2 of life skills acquisition).

We begin the practical application by teaching stress management techniques, giving particular relevance to abdominal breathing techniques (stage 3 of life skills acquisition). These were chosen due to their adaptability to different situations and because they can be helpful in managing the three dimensions of reactions. We end this topic with a group reflection on which variants are more appropriate for different situations. Participants are highly encouraged to train these techniques at least three times per day (in the morning, when they wake up, after lunch, and at night, before going to sleep). We underscore the importance of training in order to achieve automatization; when this happens, they can use the technique at any time, even standing up, without other people noticing (stage 4 of life skills acquisition).

Part 4 ends with the development of the individual stress management plan (stage 3 of life skills acquisition). We ask participants to think about their own stress management plan and how they can promote positive reactions to deal with their stress event. Moreover, it allows this knowledge to be transferred to other situations—regardless of the scenario, the same principles apply (stage 4 of life skills acquisition).

### 2.8 Part 5: the stress plan

The stress plan is arguably the most crucial part of the Pro**•**Stress program. Participants apply all the knowledge they learned to formulate a plan to deal with a specific stressor in their lives; for the case of athletes, they choose a stressor related to their sport activity. The plan is introduced in stages throughout the program—we begin its development after Part 2 (stressors), we take it up again after Part 3 (cognitive appraisal), and we finish after Part 4. Table 6 includes the stress plan structure.

**Table 6:** Stress plan structure.

Pro•Stress plan					
Section 1 | Stressor					
Stressor (please identify briefly the source of stress)…					
Section 2 | Identification of the stressor					
(Mark with a cross the dimensions that are present in your stressor)					
Stressor characteristics	1	2	3	4	5
	Novelty	Unpredictability	Complexity	Ambiguity	Uncertainty
Cognitive appraisal	1	2	3	4	5
	High or low importance	High threat	Low challenge	Low coping	Low control
Reactions to stressor	1	2		3	
	Negative psychological reactions (e.g., pessimism, disbelief, etc.)	Negative physiological/emotional reactions (e.g., increased heart rate, sweating, stomach and intestinal problems, sadness, anxiety, etc.)		Negative behavioral reactions (e.g., impulse to act, avoidance of the situation, change in daily routines, etc.)	
Section 3 | Stress management plan					
*My plan* (please identify your strategies to manage the stressor and, if appropriate, organize the use of strategies according to their temporal occurrence.)…					

Firstly, participants are encouraged to list stressors in their lives. From this list, they are asked to choose one event—preferably something that is important to them, but that they feel comfortable sharing with the group. Although our team can guarantee confidentiality, we advise participants to refrain from sharing identifying details. With the stressor firmly in mind, participants characterize it according to its characteristics (novelty, unpredictability, complexity, ambiguity, and uncertainty), which helps guide their strategies. This stage is first implemented in their work teams, under our guidance, but with participants strongly encouraged to discuss among themselves, to share their stressors and possible strategies to deal with them (stage 2 of life skills acquisition). Then, we discuss all the plans in large-group and each participant briefly describes their stressor, its characteristics, and strategies to include in their plan. Under our guidance, participants begin to shape their individual stress plans. The same procedure is followed regarding cognitive appraisal and stress reactions.

By this point, all participants have a set of loose strategies that can help them manage stress. We then attempt to organize them in a coherent plan, typically by ordering them in three stages: pre-stressor, during the stressor, and after the stressor.

When all participants achieve a somewhat stable plan, the last sessions are dedicated to its training (stages 3 and 4 of life skills acquisition). The procedure is as follows:

- Participant A briefly describes their stressor to the whole class, without revealing their plan. Others may ask clarifying questions.
- Participant A’s team meet in a room to fine tune the plan. At the same time, the two other teams are in separate rooms: one team will challenge Participant A’s plan and are preparing their strategy. The third team will observe the interaction, looking out for the characteristics of a good plan: observable, controllable, adequate, realistic, and enthusiastic.
- All participants return to the main room. Participant A has 15 min to apply their plan in role-playing as the other team challenges it.
- After training, everyone gets a turn—we hear from Participant A, how they felt, which challenges they encountered, and how they can improve their plan. The “challenging” team also explain their strategy and comment on Participant A’s performance. The observers also comment on the training and indicate specific examples of the five characteristics of a good plan. Finally, the monitors comment on the training, the specific strategies used, how they were applied, and any other topics that help Participant A improve their plan.

The plan’s formulation and training constitute precious opportunities to foster automatization. Moreover, all participants are challenged to keep revising and improving their plan according to their real-life situations. This is the first step to achieve transference: if people are capable of devising and implementing a stress management plan to one situation, they have the tools and skills to create a different plan for a different situation and can then say they truly master the stress management skill.

### 2.9 Testing the program’s efficacy

This program has yet to be tested empirically, although studies are currently taking place to do so, namely, Quasi-Experimental Studies are being undertaken with intervention and control groups of athletes and other different populations in different contexts with very promising results—measures before and after intervention appear to point to its efficacy in increasing participants’ skills in managing stress, thereby promoting a positive adaptation to stress. Despite these preliminary encouraging results yet to be published, it is now crucial to test the efficacy of the Pro**•**Stress program by comparing the level of the life skill in experimental and control groups before and after the intervention. Thus, studies to test the program should include intervention and control groups, and researchers need do adopt the most appropriate methodology to collect data (e.g., Randomized Controlled Trials, Non-Randomized Controlled Trial, Quasi-Experimental Study, Controlled Before-and-After Study). Data collection for both groups (intervention and control groups) should occur in three moments (Pre, Post, and Follow-up intervention typically collected two to four months after Post-evaluation; see Section 2.2. of this paper). This does not prevent researchers to adopt long-term follow-up studies to better monitor the effective changes produced by the program along the sports season and the athletes’ career. Additionally, research can also be designed to test the efficacy of the program across gender, age, sport disciplines and sport types, because the experience of stress can be distinct across athletes. Importantly, this paper focused on athletes, but other individuals involved in sports are also exposed to significant levels of stress, as is the case of coaches and officials. Despite all these particularities of sports context, the background to implement and evaluate the program is similar, meaning that researchers only make the necessary adjustments to the specific target they are intervening on. Finally, the program can be tested for differential effects when it is used as a compact intervention (all parts in a row) or delivered each part separately; it can also be tested in a “long” versus “short” version, because all the activities are delivered with an integral logic, meaning that monitors can select the most appropriate ones for their participants.

## 3 Conclusion

The Pro**•**Stress program is a stress management program based on the Interactive Model of Human Adaptation to Stress (IMHAS; Gomes, 2014) that aims to impart and improve stress management skills in people of all ages and backgrounds. Its strong theoretical background frames the intervention, thereby strengthening its application. Concomitantly, the program focuses heavily on the practical aspects of adaptation to stress, thus encouraging participants to reflect on the processes underlying the experience of stress while at the same time guiding specific applied strategies to deal with it. Importantly, the fact that participants are expected to devise their own stress management plan allows for the individualization of life skills learning and training. The program also encourages training through role-playing exercises, which strengthens participants’ performance and self-confidence and allows them to more easily apply their plan in real-life contexts. Above all, the focus is on teaching participants the tools to analyze and manage their stressors, thereby empowering them to repeat the process for any other stressor they may face—this will achieve the last stage of life skills acquisition, transference. Nevertheless, before having final data demonstrating the effectiveness of the program, all the information about this program should be taken with caution, since it is necessary to wait for final data of research testing to determine how useful the program is in improving the athletes’ ability to evaluate and cope with stress. Significantly, future research should clarify the potential impacts produced by the intervention in distinct areas of the athletes’ functioning (i.e., event outcomes of the IMHAS model), as is the case of psychological variables like mental energy (Yildiz et al., 2025) and mental toughness (Kesler et al., 2026), the mental health and wellbeing of athletes (Prior et al., 2024), and even sports performance (Lochbaum et al., 2022). In sum, the Pro**•**Stress program aims to respond to the ever-growing challenge of managing stressful events by relying on a strong theoretical background and a robust applied methodology.
